# Dynamics, morphogenesis and convergence of evolutionary quantum Prisoner's Dilemma games on networks

**DOI:** 10.1098/rspa.2015.0280

**Published:** 2016-02

**Authors:** Angsheng Li, Xi Yong

**Affiliations:** 1 State Key Laboratory of Computer Science, Institute of Software, Chinese Academy of Sciences, Beijing 100190, People's Republic of China; 2 School of Computer Science, University of Chinese Academy of Sciences, Beijing 100190, People's Republic of China

**Keywords:** networks, game, entanglement

## Abstract

The authors proposed a quantum Prisoner's Dilemma (PD) game as a natural extension of the classic PD game to resolve the dilemma. Here, we establish a new Nash equilibrium principle of the game, propose the notion of convergence and discover the convergence and phase-transition phenomena of the evolutionary games on networks. We investigate the many-body extension of the game or evolutionary games in networks. For homogeneous networks, we show that entanglement guarantees a quick convergence of super cooperation, that there is a phase transition from the convergence of defection to the convergence of super cooperation, and that the threshold for the phase transitions is principally determined by the Nash equilibrium principle of the game, with an accompanying perturbation by the variations of structures of networks. For heterogeneous networks, we show that the equilibrium frequencies of super-cooperators are divergent, that entanglement guarantees emergence of super-cooperation and that there is a phase transition of the emergence with the threshold determined by the Nash equilibrium principle, accompanied by a perturbation by the variations of structures of networks. Our results explore systematically, for the first time, the dynamics, morphogenesis and convergence of evolutionary games in interacting and competing systems.

## 1. Introduction

The Prisoner's Dilemma (PD) game is one of the well-known games, having implications in a wide range of disciplines. In a PD game, two players simultaneously decide their strategy, *C* (cooperator) or *D* (defector). For mutual cooperation, both players receive a reward *R* and receive punishment *P* upon mutual defection. If one cooperates and the other defects, then the cooperator gains the lowest pay-off *S* and the traitor gains temptation *T*. The pay-off rank for the PD game is given by *T*>*R*>*P*>*S*. In a PD game, the best strategy for both players is to defect regardless of the other's decision, in which case the pay-offs of the players are minimized.

Real-world games are played in a system characterized by a graph in which the nodes are players and the edges represent the games played by the two endpoints of each of the edges. The games in a graph are evolving by rounds. In this case, the emergence of cooperation of the evolutionary games on a graph implies a maximal global pay-off that evolves from minimal local pay-offs of the graphs. It plays an essential role in the organizations of graphs of systems such as the biosphere and human society.

Evolutionary game theory is devoted to understanding the emergence of cooperation and the cooperative behaviours in games in Nature and society. Nowak & May [1] showed that spatial reciprocity (or spatial structure) is a mechanism promoting cooperation by introducing a weak version of the PD game in which the pay-offs are chosen as *R*=1, *P*=*S*=0 and *T*=*b*, for *b*>1. Nowak [2] introduced five simple rules for cooperation in evolutionary games.

Social structures have been found to play a role in the emergence of cooperation in evolutionary games (see [3–5]). It has also been shown that heterogeneity in networks plays a role in the emergence of cooperation in evolutionary games on networks [6–9]. This property has also been found in other games [9–13]. On the other hand, cooperation is unlikely to emerge in the evolutionary PD games on homogeneous networks. Reciprocity and rewarding fitness were found to play an essential role in the emergence of cooperation in evolutionary games on networks [14–17]. Different updating strategies may also play a role in the emergence of cooperation in evolutionary games on networks [18]. In all of these studies, the PD game is the weak version defined by Nowak & May [1].

In 1999, Eisert *et al.* [19] proposed a quantum game in which each player chooses a strategy from a unitary operator. Recently, the emergence of cooperation of evolutionary quantum games has become an interesting new topic [20–23]. The current authors [24] proposed a quantum PD game by introducing entanglement into the games and by introducing a new strategy of super-cooperator based on the weak version PD game in Nowak & May [1]. It was shown that, for appropriately large entanglement, super-cooperation quickly emerges in the evolutionary quantum PD games on random graphs generated by the Erdös–Rényi (ER) model [25], and the small-world model [26].

For the full version of the PD game, we consider a normalized version: it is usually defined by a parameter *r*, in which case the reward *R*=1, the temptation *T*=1+*r*, the punishment *P*=0 and the lowest ‘sucker's’ pay-off *S*=−*r*. The current authors [27] proposed a quantum PD game based on both the full and normalized PD game. It was shown that both versions of the quantum PD games have a new Nash equilibrium principle, and that for heterogeneous networks of the preferential attachment (PA) model [7], and for appropriately large entanglement, super-cooperation quickly emerges in evolutionary quantum PD games on the networks. This is the first time that evolutionary games on networks have been studied for the normalized full PD games. We note that it is unlikely that cooperation emerges in evolutionary PD games on networks for either the full or normalized full version of PD games.

Alan Turing [28] proposed a mathematical model to understand morphogenesis in biology: the gastrulation phase of embryonic development, i.e. the process whereby dappling effects arise on animal coats, and phyllotaxy, i.e. the arrangement of leaves on plant stems. However, this process can be extended to the general question: How does morphogenesis occur? or How do structures arise from an initially random configuration? Barmpalias *et al.* [29] established such a theory by using the Schelling segregation model [30].

In the present article, we investigate the equilibria, dynamics, morphogenesis, convergence and phase transition of evolutionary games on the networks of classical models [7,25,26] for the normalized full version of the PD game introduced in [27]. Our results establish a new theory of the convergence and phase transition of evolutionary games in networks.

## 2. Quantum Prisoner's Dilemma games

Quantum entanglement exists in many physical and chemical processes, and it may even exist in some biological processes. The current authors [24,27] introduced a quantum PD game to resolve the classical PD in Nature and society. Our quantum PD game naturally extends the classical PD game by using a quantum entanglement to measure the complex relationship between players that certainly exists in real-world games.

Our game consists of two players, Alice and Bob. We assume that Alice and Bob have an entanglement measured by *γ*∈[0,*π*/2], and that if both Alice and Bob choose strategies *C* or *D* only, then the game between Alice and Bob is the same as a classical PD game.

Initially, the players share an entangled state of the form $|\psi_{0}\rangle =\hat{J}|0\rangle \times |0\rangle$, where $\hat{J}$ is an entangling operator. The degree of entanglement is measured by a real number *γ*∈[0,*π*/2]. Next, the players are allowed to independently choose a unitary operation $\hat{U}$ of the following form:

$$\hat{U}(\theta ,\phi )=\left(\begin{array}{cc} \frac{e^{i\phi }\cos\theta }{2} & \frac{\sin\theta }{2}\\ -\frac{\sin\theta }{2} & \frac{e^{-i\phi }\cos\theta }{2} \end{array}\right),$$

for *θ* and *ϕ* ranging from 0 to *π* and from 0 to *π*/2, respectively.

In our game, each player has only three strategies: *C*, *D* and *Q*, given by $C=\hat{U}(0,0)$, $D=\hat{U}(\pi ,0)$ and $Q=\hat{U}(0,\pi /2)$. *C* and *D* correspond to cooperation and defection in the classical PD game, respectively, and *Q* is called *super-cooperation*.

**Figure 1.**
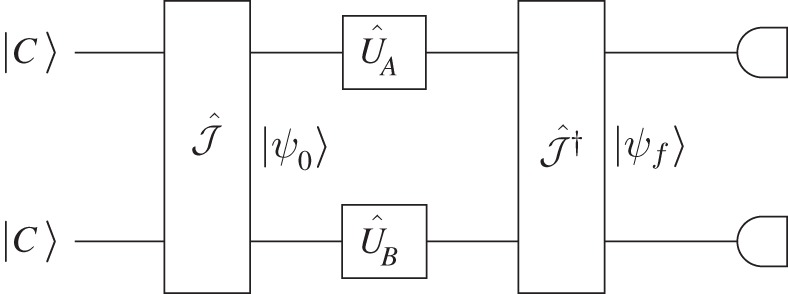
The quantum device for a two-player game.

The qubits from the players A and B are sent to a quantum machine in figure 1 that applies a unitary operator ${\hat{J}}^{\;†}$ to the qubits, yielding a final state (see [19]).

Finally, a joint quantum measurement is then applied to the final state to determine the expectation value of the individual pay-offs of the players based on the pay-offs of the classical PD game. The pay-off matrix of the row players is thus given in table 1.

**Table 1.** GPD_N_. The expectation pay-off matrix of our quantum PD game—the normalized version. The value in the matrix is the pay-off of the row strategy.

	*C*	*D*	*Q*
*C*	1	−*r*	$\cos^{2}\gamma$
*D*	1+*r*	0	$(1+r)\cos^{2}\gamma -r\sin^{2}\gamma$
*Q*	$\cos^{2}\gamma$	$-r\cos^{2}\gamma +(1+r)\sin^{2}\gamma$	1

The game in table 1 is a normalized full version of the PD game. We use QPD_N_ to denote the game.

According to table 1, if *γ*=0, then *Q*=*C* and the game is the classical PD game; and if *γ*=*π*/2, then the game is the quantum game proposed by Eisert *et al.* [19]. Therefore, our game is a natural extension of both the classical and quantum PD games. It allows us to analyse the role of varying entanglement in the PD game. The fundamental principle of our game is the following phase-transition phenomenon.

*Nash equilibrium principle of the quantum PD game—normalized version*. (1) If $\gamma >\arccos\sqrt{\left(1+r)/(1+2r\right)}$, then (*Q*,*Q*) is the unique Nash equilibrium which achieves the maximal pay-offs; and (2) if $\gamma <\arccos\sqrt{\left(1+r)/(1+2r\right)}$, then (*D*,*D*) is the unique Nash equilibrium which achieves the maximal pay-offs.

For (1). Assume $\gamma >\arccos\sqrt{\left(1+r)/(1+2r\right)}$. In this case, Alice may maximize her minimum pay-off (i.e. the guaranteed pay-off whatever Bob chooses) by taking a *Q* strategy; and if Alice takes *Q*, then the maximum pay-off of Bob is $\max\{\cos^{2}\gamma ,(1+r)\cos^{2}\gamma -r\sin^{2}\gamma ,1\}=1$, which is achieved if Bob takes *Q*. For (2). Assume $\gamma <\arccos\sqrt{\left(1+r)/(1+2r\right)}$. In this case, Alice may maximize her minimum pay-off by taking a *D* strategy; and if Alice takes *D*, then the maximum pay-off of Bob is $\max\{-r,0,-r\cos^{2}\gamma +(1+r)\sin^{2}\gamma \}=0$, which is achieved if Bob takes *D*.

The Nash equilibrium principle ensures that there is a phase transition from *D* to *Q*, determined by the critical point $\arccos\sqrt{\left(1+r)/(1+2r\right)}$, and that a player Alice may force her opponent player Bob to cooperate by choosing a *Q* strategy, if the entanglement degree between the two players is above the phase-transition point. More importantly, we note that, although the law behind the game in table 1 is quantum mechanics, the game itself can be regarded as a classical extension of the well-known PD game. This solves the classic PD by assuming that there is a complex relationship between the players, and that the relationship between the players is measured by a degree of entanglement. The new game hence allows us to analyse the interacting and competing systems in both micro- and macro-worlds.

## 3. Convergence of cooperation in evolutionary games on networks

Evolutionary games is a many-body extension of the game on the network of interacting agents. The existing literature suggests a number of ways to prompt the emergence of cooperation. However, there is no principal characterization for even just the emergence of cooperation. The stronger notion of convergence is simply missing in the current state of the art. Our game may make a change. We observe that if the Nash equilibrium principle in §2 holds for evolutionary games on networks, then we are able to study the convergence of cooperation in evolutionary games.

We first introduce some basic notions and terminologies. Given a network *G*=(*V*,*E*), and a two-player game with strategies *C*, *D* and possibly other *X*'s—say, the PD game—an evolutionary game on *G* proceeds as follows:

(1) At step 0, for every node *v*∈*V* , we pick randomly and uniformly a strategy *C* or *D* or *X* (if any); for *v*, we use *s*(*v*)[0] to denote this strategy.
(2) At step *t*, suppose that the strategy of *v* at step *t*, *s*(*v*)[*t*], is defined for every node *v* in *V* . Then: (i) for every *v*∈*V* , let *P*(*v*)[*t*] be the total pay-off of *v* obtained from the games of *v* with all its neighbours in *G* at step *t*; (ii) for every *v*∈*V* , randomly and uniformly choose a neighbour *u* of *v*—if *P*(*u*)[*t*]>*P*(*v*)[*t*], then with a probability determined by *P*(*u*)[*t*] and *P*(*v*)[*t*], set s(v)[t+1]←s(u)[t], and (iii) go back to step (ii).

**Remark**

In this paper, the updating strategy in (2) above is realized by the Fermi rule in equation (4.1). However, step (2) does not require the specific updating rule. The motivation for this difference is to emphasize that there are other reasonable updating strategies, and that the concepts developed here may be developed for the evolutionary games using other reasonable updating strategies.

We use E to denote an evolutionary game defined as above. In the evolutionary games above, for every *t*, let $\rho_{G}^{E}(C)[t]$ be the fraction of nodes *v* which share strategy *C*. Similarly, we can define $\rho_{G}^{E}(X)[t]$ for the strategy *X*≠*C*.

We say that cooperation emerges in evolutionary games on *G*, if there is a large constant *δ*, $>\frac{3}{4}$, say, and a step *T* such that, for every *t*≥*T*, the following property holds:

3.1 $$E[\rho_{G}^{E}(C)[t]]\geq \delta ,$$

where E is a random evolution of the games on *G*, and $E[\rho_{G}^{E}(C)[t]]$ is the expectation of $\rho_{G}^{E}(C)[t]$.

Let M be a model of networks. We say that cooperation emerges in evolutionary games on the networks of model M, if for every type of M there is a large constant *δ* and a step *T* such that, for every *t*≥*T*, the following property holds:

3.2 $$E[\rho_{G}^{E}(C)[t]]\geq \delta ,$$

where *G* is the network of the given type generated by M, E is a random evolution of the games on *G* and $E[\rho_{G}^{E}(C)[t]]$ is the expectation of $\rho_{G}^{E}(C)[t]$.

Similarly, we can define the emergence of other strategies in evolutionary games on networks.

By definition, the notion of emergence is determined by the large constant *δ* and the step *T*. The larger *δ* is, the better the quality of the emergence is. If *T* is small, then we say that it is a quick emergence.

Emergence of cooperation requires that $E[\rho_{G}^{E}(C)[t]]\geq \delta$ holds. However, this does not guarantee any global cooperation of evolutionary games on the networks. For this reason, we propose the notion of convergence.

Given a network *G*, and a two-player game with strategies that include both *C* and *D*, the PD, for example, we say that cooperation converges in evolutionary games on *G*, if there is a large constant *δ*, a step *T*, such that, for every *t*≥*T*, the following property holds:

3.3 $$Pr[\rho_{G}^{E}(C)[t]\geq \delta ]\geq 1-o(1),$$

where the probability is over the random evolution E.

Let M be a model of networks. We say that cooperation converges in evolutionary games on networks of model M, if for any type of the model there is a large constant *δ* and a step *T* such that, for every *t*≥*T*, the following property holds:

3.4 $$Pr[\rho_{G}^{E}(C)[t]\geq \delta ]=1-o(1),$$

where the probability is over the random choice of network *G* of the type by model M, and over the random evolution E on *G*, 1−*o*(1) is a function with limit 1 as the size *n* of *G* goes to infinity.

We may define the notion of convergence of other strategies similarly.

By definition, the fundamental issues of convergence of cooperation defined above are (i) the *δ* should be large, or ≈1 or even =1; (ii) the probability $Pr[\rho_{G}^{E}(C)[t]\geq \delta ]$ is nearly equal to 1; and (iii) the number *T* is small.

The three properties above ensure that a global cooperation is almost surely guaranteed to quickly occur in any evolution of the games on the networks. Therefore, the conditions of convergence in equations (3.3) and (3.4) are much stronger than the condition $E[\rho_{G}^{E}(C)[t]]\geq \delta$ of emergence.

We emphasize that our experiments for convergence are essentially different from the existing experiments of emergence. The differences are as follows: (I) for emergence, the equilibrium frequency, *ρ*(*C*) say, is the average cooperation ratio of the last *N* steps of each of the *M* evolutions of each of the *K* networks of the same type for some natural numbers *N*, *M* and *K*; and (II) for convergence, the equilibrium frequency, *ρ*(*C*) say, is the least cooperation ratio of the last *N* steps of each of the *M* evolutions of each of the *K* networks of the same type for some natural numbers *N*, *M* and *K*.

To the best of our knowledge, all previous studies implemented only type I experiments. Our experiments of convergence are of type II.

## 4. Dynamics and morphogenesis of evolutionary quantum games

To understand the evolutionary quantum PD games, we first investigate the dynamics of the evolutionary games on networks. Given a network *G*, an evolution of the games on *G* proceeds by steps. Initially, every node *i* chooses a strategy *C*, or *D* or *Q* uniformly and randomly with equal probability. The pay-off of node *i* is the total pay-offs of *i* from the games between *i* and all its neighbours. During the games, the updating probability of a node to adopt the strategy of a reference node is determined by the Fermi function as follows. For each node *i*, *i* randomly and uniformly picks a neighbour *j*. Then the probability that node *i* adopts the last strategy of node *j* is defined by

4.1 $$P=\frac{1}{1+\exp[-(P_{j}-P_{i})/T]},$$

where *P*_*i*_, *P*_*j*_ are the current total pay-offs of nodes *i* and *j*, respectively, and *T* is a parameter representing the noise of the updating strategy. In all of our experiments, we set *T*=0.04. We use $E^{G}$ to denote an evolution of the games on *G*.

**Figure 2.**
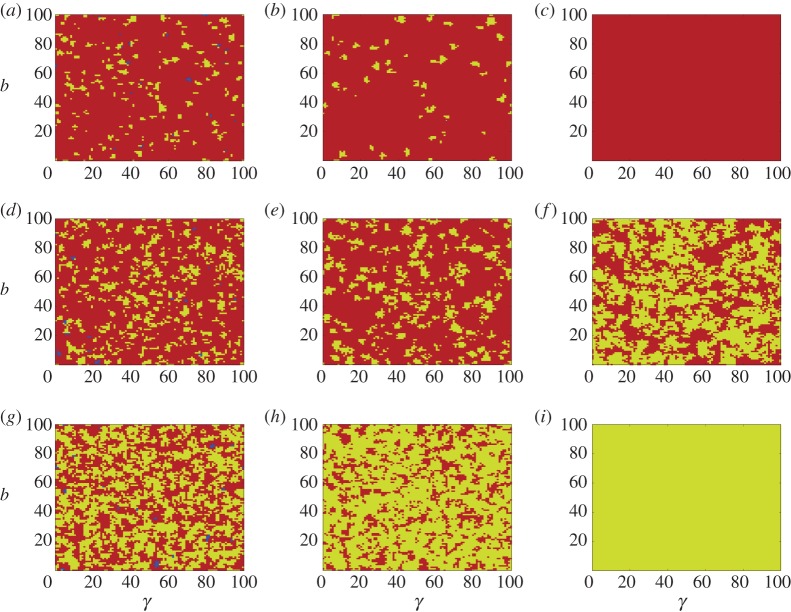
Dynamics of the evolutionary quantum PD games on a grid of 100×100. The *C*-, *D*- and *Q*-strategy nodes are coloured blue, red and yellow, respectively. For *r*=0.6, and $\gamma_{0}=\arccos\sqrt{\left(1+r)/(1+2r\right)}$. (*a*–*c*) The configurations of the games for *γ*=*γ*_0_−5° by the end of steps 5, 10 and 100, respectively. (*d*–*f*) The configurations of the games for *γ*=*γ*_0_ by the end of the same steps 5, 10 and 100, respectively. (*g*–*i*) The configurations of the games for *γ*=*γ*_0_+5° by the end of steps 5, 10 and 100, respectively.

In figure 2*a*–*i*, we depict the dynamics of the evolutions of the quantum PD games. In this experiment, the graph is a 100×100 grid. For *r*=0.6, and $\gamma_{0}=\arccos\sqrt{\left(1+r)/(1+2r\right)}$, the games in figure 2*a*–*i* have entanglement degrees *γ*_0_−5°, *γ*_0_ and *γ*_0_+5°, respectively. The *C*-, *D*- and *Q*-strategy nodes are coloured blue, red and yellow, respectively. Figure 2*a*,*d*,*g* is the distributions of the strategies by the end of step 5. Figure 2*b*,*e*,*h* is the distributions of the strategies by the end of step 10. Figure 2*c*,*f*,*i* is the distributions of the strategies by the end of step 100.

In the experiments described in this paper, the networks and the entanglement degree *γ* are both simulated on a classical system. We remark that it is a challenge in physics to prepare a many-body quantum game system with given entanglement degree *γ*.

By observing figure 2, we have the following results:

(1) For *γ*=*γ*_0_−5°. We have: (1a) by figure 2*a*, at the end of step 5, the cooperators almost vanish, super-cooperators form a number of small pieces that are evenly distributed, and the defectors occupy a number of large areas; (1b) by figure 2*b*, at the end of step 10, the cooperators vanish, the super-cooperators form a small number of small pieces, and the defectors almost occupy the graph; and (1c) by figure 2*c*, at the end of step 100, the defectors occupy the whole graph.
(2) For *γ*=*γ*_0_. We have: (2a) by figure 2*d*, at the end of step 5, the cooperators almost vanish, both the defectors and the super-cooperators form a number of small communities; (2b) by figure 2*e*, at the end of step 10, the *C*-strategy nodes vanish, and the *D*- and the *Q*-strategy nodes form similarly a large number of small communities which are eventually distributed in the graph; and (2c) by figure 2*f*, at the end of step 100, the *D*- and *Q*-strategy nodes both form a large number of small communities that are evenly distributed in the grid.
(3) For *γ*=*γ*_0_+5°. We have: (3a) by figure 2*g*, at the end of step 5, the cooperators almost vanish, the defectors form small communities, and the super-cooperators form small communities; (3b) by figure 2*h*, at the end of step 10, the cooperators vanish, the defectors form small communities, and the super-cooperators form large areas; and (3c) by figure 2*i*, at the end of step 100, the super-cooperators occupy the whole graph.
(4) By figure 2*a*,*d*,*g* in any case, the cooperators almost vanish in the evolutions in a few initial steps.
(5) By figure 2*b*,*e*,*h*, the cooperators vanish in a few initial steps, and, after the *C*-strategy nodes have been conquered, the competition is between the *D*- and the *Q*-strategy nodes.
(6) By figure 2*c*,*f*,*i*, the conqueror strategy is determined by the entanglement *γ*. If *γ* is slightly smaller than $\gamma_{0}=\arccos\sqrt{\left(1+r)/(1+2r\right)}$, then the defectors quickly conquer the network; if *γ* is slightly larger than $\gamma_{0}=\arccos\sqrt{\left(1+r)/(1+2r\right)}$, then the super cooperators quickly conquer the network; and if *γ* is close to $\gamma_{0}=\arccos\sqrt{\left(1+r)/(1+2r\right)}$, then the defectors and super-cooperators coexist, in which case both the defectors and super-cooperators form small communities that are evenly distributed in the network.

Results (1)–(6) explore the following *dynamics principle of evolutionary quantum PD games*: (1) (cooperators find it hard to survive in homogeneous networks) in any case, the cooperators are always conquered in the initial steps of the evolutionary games; (2) (individuals of the same strategy form communities) during the procedure of the evolutionary games, individuals of the same strategy always merge together into communities; (3) (the winning strategy is expanding) individuals of the winning strategy will form larger and larger communities until they occupy or dominate the network; (4) (the non-winning strategy is shrinking) individuals of the non-winning strategy can only form small pieces during the procedure of the evolutionary games, until they are eliminated, if there is a winning strategy of the games; (5) (defectors win) if *γ* is slightly less than $\gamma_{0}=\arccos\sqrt{\left(1+r)/(1+2r\right)}$, then the defectors quickly form large areas, and occupy the graph; at the same time, the cooperators quickly vanish, and the super-cooperators form small pieces in early evolutions and quickly vanish from the network; (6) (super-cooperators win) if *γ* is slightly larger than $\gamma_{0}=\arccos\sqrt{\left(1+r)/(1+2r\right)}$, then the cooperators quickly vanish, the defectors form fewer and fewer small communities, and the super-cooperators form larger and larger areas and quickly occupy the graph; (7) (*D* and *Q* coexist) if $\gamma =\arccos\sqrt{\left(1+r)/(1+2r\right)}$, then the cooperators quickly vanish, and each of the defectors and super-cooperators forms approximately the same number of small communities which are evenly distributed in the graph; (8) (equilibrium game both defectors and super-cooperators) the defectors and super-cooperators coexist only in a narrow belt along the curve $\gamma =\arccos\sqrt{\left(1+r)/(1+2r\right)}$ in evolutionary quantum PD games on homogeneous networks; and (9) (morphogenesis by evolutionary games) during the evolutionary games, individuals of the same strategy always merge together to form connected regions. This phenomenon may well explain the reason why people who live in different regions have different cultures and philosophies.

Properties (1)–(9) above very well explore the dynamics and social morphogenesis of evolutionary quantum PD games on grid graphs, hence on homogeneous networks and, perhaps, on general networks.

## 5. Mean-field theory of evolutionary quantum PD games on networks

To better understand the dynamics in §4 and the game in table 1, we propose a mean-field theory of the evolutionary games. Given a network *G*, and the evolutions of the games on *G*, we define *ρ*_*C*_,*ρ*_*D*_ and *ρ*_*Q*_ to be the probability that a node takes strategy *C*, *D* and *Q*, respectively. In fact, the notations are functions of step *t* of the evolutionary games. During the procedure of the evolutions, we have that *ρ*_*C*_+*ρ*_*D*_+*ρ*_*Q*_=1. In particular, in step 0 of the evolutionary games, each of *ρ*_*C*_, *ρ*_*D*_ and *ρ*_*Q*_ is $\frac{1}{3}$ in our experiments. Note that this is not necessary. For both our theory and experiments, the initial distribution of *ρ*_*C*_, *ρ*_*D*_ and *ρ*_*Q*_ can be arbitrarily given with some trivial conditions such as none of the three probabilities is more than $\frac{1}{2}$.

By the definition of the game in table 1, we have that the pay-offs of the *C*-, *D*- and *Q*-strategy nodes are defined by *P*_*C*_, *P*_*D*_ and *P*_*Q*_, respectively, as

5.1 $$\begin{array}{rl} P_{C} & =E[\rho_{C}+\cos^{2}\gamma \cdot \rho_{Q}-r\cdot \rho_{D}], \end{array}$$

5.2 $$\begin{array}{rl} P_{D} & =E[(1+r)\cdot \rho_{C}+((1+2r)\cos^{2}\gamma -r)\cdot \rho_{Q}] \end{array}$$

5.3 $$\begin{array}{rl} \text{and}\;P_{Q} & =E[\cos^{2}\gamma \cdot \rho_{C}+\rho_{Q}+(1+r-(1+2r)\cos^{2}\gamma )\cdot \rho_{D}], \end{array}$$

where *E*[*X*] is the expectation of random variable *X*.

By definition of the game, the derivative of *ρ*_*C*_ is the following *master equation*:

5.4 $${\dot{\rho }}_{C}=-\rho_{C}[W(C\to D)+W(C\to Q)]+\rho_{D}\cdot W(D\to C)+\rho_{Q}\cdot W(Q\to C),$$

where W(X→Y) is the probability that strategy *X* transfers to strategy *Y* .

By the Fermi rule of the updating strategy of evolutionary games, we have that

5.5 $${\dot{\rho }}_{C}=-\rho_{C}\cdot \rho_{D}\cdot \tanh\left(\frac{P_{D}-P_{C}}{2T}\right)-\rho_{C}\cdot \rho_{Q}\cdot \tanh\left(\frac{P_{Q}-P_{C}}{2T}\right).$$

By the same argument, we have that

5.6 $${\dot{\rho }}_{Q}=\rho_{Q}\cdot \rho_{C}\cdot \tanh\left(\frac{P_{Q}-P_{C}}{2T}\right)+\rho_{Q}\cdot \rho_{D}\cdot \tanh\left(\frac{P_{Q}-P_{D}}{2T}\right)$$

and

5.7 $${\dot{\rho }}_{D}=\rho_{D}\cdot \rho_{C}\cdot \tanh\left(\frac{P_{D}-P_{C}}{2T}\right)+\rho_{D}\cdot \rho_{Q}\cdot \tanh\left(\frac{P_{D}-P_{Q}}{2T}\right).$$

To understand equations (5.5)–(5.7), we analyse *P*_*Q*_−*P*_*D*_, *P*_*Q*_−*P*_*C*_ and *P*_*D*_−*P*_*C*_.

By equations (5.1)–(5.3), we have the following equations:

5.8 $$\begin{array}{rl} P_{Q}-P_{D} & =E[(2\rho_{C}-(1+2r))\cos^{2}\gamma -(1+r)(2\rho_{C}-1)] \end{array}$$

5.9 $$\begin{array}{rl} P_{Q}-P_{C} & =E[(1+2r)\rho_{D}+(\rho_{Q}-\rho_{C})(1-\cos^{2}\gamma )] \end{array}$$

5.10 $$\begin{array}{rl} \text{and}\;P_{D}-P_{C} & =E[(1+r)\rho_{Q}\cos^{2}\gamma +r(1-2\rho_{Q})]. \end{array}$$

At first, we analyse the cooperation ratio *ρ*_*C*_. By equation (5.9), if *ρ*_*Q*_≥*ρ*_*C*_, then *P*_*Q*_−*P*_*C*_>0, which holds from the beginning of the evolution of the quantum PD games. By equation (5.10), initially, we have that $\rho_{Q}<\frac{1}{2}$, so that *P*_*D*_−*P*_*C*_>0. Therefore, by equation (5.4), during the early stages of the evolutionary quantum PD games, the two properties of *P*_*Q*_−*P*_*C*_>0 and *P*_*D*_−*P*_*C*_>0 ensure that ${\dot{\rho }}_{C}<0$. This property implies the following property.

*Cooperators vanishing law*. Statistically, *ρ*_*C*_ goes to 0 during the early stages of the evolutionary PD games on a network, that is, cooperators vanish in the early steps of the evolutionary games, unless the network is perturbed by random variations. Precisely, we have that the equilibrium frequency of cooperators *ρ*_*C*_ is either 0 or a small random variable *ϵ* determined by random variations.

We thus conclude that cooperators quickly vanish in the early stages of the evolutionary quantum PD games, with a slight perturbation by the structures of the networks. For this reason, we interpret the equilibrium frequency of cooperators *ρ*_*C*_ as the perturbation of evolutionary quantum PD games on a network.

Nevertheless, by the cooperators vanishing law above, we have that the perturbation *ρ*_*C*_ is either 0, or ≈0, or *ϵ* for some small number *ϵ*.

Secondly, we analyse the emergence of strategies of the evolutionary quantum PD games on networks. We have the following phase-transition phenomenon:

*Case 1*. $\gamma >\arccos\sqrt{\left(1+r)(1-2\rho_{C}\right)/\left[(1+2r)-2\rho_{C}\right]}$.

By equation (5.8), we have *P*_*Q*_−*P*_*D*_>0. By equation (5.9), we will have *P*_*Q*_−*P*_*C*_>0. By equation (5.6), we have that ${\dot{\rho }}_{Q}>0$, and that *ρ*_*Q*_ goes to 1 in the evolutionary quantum PD games on the network.

*Case 2*. $\gamma \approx \arccos\sqrt{\left(1+r)(1-2\rho_{C}\right)/\left[(1+2r)-2\rho_{C}\right]}$.

In this case, by equation (5.8), we have that *P*_*Q*_≈*P*_*D*_. Furthermore, by equations (5.6) and (5.7), we have that ${\dot{\rho }}_{Q}\approx 0$ and ${\dot{\rho }}_{D}\approx 0$.

This implies that cooperators vanish by following the cooperators vanishing law with a perturbation by structures of networks, and that defectors and super-cooperators have an equilibrium game in the evolutionary PD games on networks, in the sense that none of the defectors or the super-cooperators conquers the other.

*Case 3*. $\gamma <\arccos\sqrt{\left(1+r)(1-2\rho_{C}\right)/\left[(1+2r)-2\rho_{C}\right]}$.

By equation (5.8), we will have *P*_*D*_>*P*_*Q*_. By equation (5.10), we will have that

$$P_{D}-P_{C}>\frac{\left(1+r)\rho_{Q}+r(1+2r)(1-2\rho_{Q}\right)}{1+2r}=\frac{(1-3r^{2})\rho_{Q}+r+2r^{2}}{1+2r}\geq \frac{1+r-r^{2}}{1+2r}>0.$$

The last inequality follows from the assumption that 0<*r*≤1.

By equality (5.7), ${\dot{\rho }}_{D}>0$, which ensures that *ρ*_*D*_=1.

We emphasize that the threshold $\arccos\sqrt{\left(1+r)(1-2\rho_{C}\right)/\left((1+2r)-2\rho_{C}\right)}$ is principally $\arccos\sqrt{\left(1+r)/(1+2r\right)}$ with a slight perturbation by the equilibrium frequency of cooperators *ρ*_*C*_, that the threshold $\arccos\sqrt{\left(1+r)/(1+2r\right)}$ is given by the Nash equilibrium principle for our game in table 1, and that the perturbation *ρ*_*C*_ is determined by variations of the evolutionary games and by the cooperators vanishing law. (Equally important, we note that the perturbation of the phase transition is determined by *ρ*_*C*_, the cooperation frequency. Our vanishing law says that if the network is homogeneous, then *ρ*_*C*_ goes to 0. However, for some networks, *ρ*_*C*_ may exist, which implies an emergence of cooperation of the classical games in the network.)

The cooperators vanishing law and the phase-transition principle show the dynamics in §4, and imply that the Nash equilibrium principle in §2 holds for the many-body extension of the game to many networks.

## 6. Convergence principle

In this section, we investigate the convergent strategy of evolutionary quantum PD games on homogeneous networks.

For an evolution E on *G*, for a strategy *X*∈{*C*,*D*,*Q*}, and *t*, we use $\rho_{G}^{E}(X)[t]$ to denote the fraction of vertices *v* in *G* which have strategy *X* during step *t*. We say that *super-cooperation converges in the evolutionary PD games on *G**, if there exist a small number *T*, and a large constant *ρ*_0_, larger than $\frac{4}{5}$ say, or even ≈1, such that, almost surely, i.e. with probability 1 as the size *n* of the network goes to infinity, the following property holds: for any evolution E of the PD games in *G*, and for every *t*≥*T*,

6.1 $$\rho_{G}^{E}(Q)[t]\geq \rho_{0}.$$

Similarly, we can define the convergence for the *D*- and *C*-strategy nodes.

To design the experiments of convergence, we introduce some notations. Given a model of networks, M say, and a type, we generate *N* networks of the model with the same type. Suppose that *G*_1_,*G*_2_,…*G*_*N*_ are the networks constructed by this way. For each network *G*_*i*_, we implement *M* evolutions, denoted by $E_{1}^{\left(i\right)},E_{2}^{\left(i\right)},\ldots E_{M}^{\left(i\right)}$. Each evolution $E_{j}^{\left(i\right)}$ on *G*_*i*_ proceeds as follows: for every node *v*, we randomly and uniformly define the initial strategy *s*(*v*)[0] to be one of *C*, *D* or *Q*. Then the evolutionary quantum PD games on *G*_*i*_ execute 10 000 steps. For a strategy *S*, and for each round *t*, let *ρ*_*i*,*j*_(*S*)[*t*] be the fraction of nodes which have strategy *S* during step *t*. We define

6.2 $$\begin{array}{rl} \rho_{i,j}^{S} & =\min\{\rho_{i,j}^{S}[t]\;|\;5000<t\leq 10\;000\}, \end{array}$$

6.3 $$\begin{array}{rl} \rho_{i}(S) & =\min\{\rho_{i,j}(S)\;|\;1\leq j\leq M\} \end{array}$$

6.4 $$\begin{array}{rl} \text{and}\;\rho (S) & =\min\{\rho_{i}(S)\;|\;1\leq i\leq N\}. \end{array}$$

Then *ρ*(*S*) is approximately the convergent equilibrium of strategy *S* of the networks of model M of the given type.

In our experiments, we use *ρ*(*Q*)≈1, *ρ*(*C*)≈1 and *ρ*(*D*)≈1 to denote the convergence of super-cooperation, cooperation and defection, respectively.

**Figure 3.**
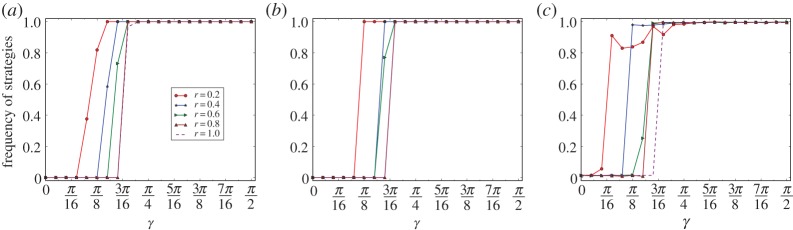
Convergence of super-cooperation. The equilibrium frequencies in the figure are the minimal ratio of super-cooperators of the last 5000 steps of 10 000 steps for 10 evolutions of each of 10 networks of the type of the models, except for the grid model for which each type has only one graph. (*a*), (*b*) and (*c*) are for the grid graphs, networks of the small-world model and the ER model, respectively.

In figure 3*a*, we depict the curves of *ρ*(*Q*) for a grid of 100×100 nodes, with 10 evolutions for *r*=0.2,0.4,0.6,0.8 and 1, respectively. In figure 3*b*, we depict the curves of *ρ*(*Q*) for networks with 10 000 nodes, and *p*=0.01 of the two-dimensional small-world model, where the curve *ρ*(*Q*) is defined by 10 networks, each of which runs 10 evolutions, for *r*=0.2,0.4,0.6,0.8 and 1, respectively. In figure 3*c*, we depict the curves of *ρ*(*Q*) for networks with 10 000 nodes, and $p=\frac{8}{10\;000}$ of the ER model, where the curve *ρ*(*Q*) is defined by 10 networks, each of which runs 10 evolutions, for *r*=0.2,0.4,0.6,0.8 and 1, respectively.

For the grid graphs. By observing figure 3*a*, we have the following. (1) For *r*=0.2. If *γ*>3*π*/32, then for any evolution E and any *t*>5000, the equilibrium frequency of the super-cooperators at step *t* is $\rho_{G}^{E}(Q)[t]=1$. (2) For *r*=0.4. If *γ*>*π*/8, then for any evolution E and any *t*>5000, the equilibrium frequency of the super-cooperators at step *t* is $\rho_{G}^{E}(Q)[t]=1$. (3) For *r*=0.6. If *γ*>5*π*/32, then for any evolution E and any *t*>5000, the equilibrium frequency of the super-cooperators at step *t* is $\rho_{G}^{E}(Q)[t]=1$. (4) For *r*=0.8. If *γ*>3*π*/16, then for any evolution E and any *t*>5000, the equilibrium frequency of the super-cooperators at step *t* is $\rho_{G}^{E}(Q)[t]=1$. (5) For *r*=1. If *γ*>3*π*/16, then for any evolution E and any *t*>5000, the equilibrium frequency of the super-cooperators at step *t* is $\rho_{G}^{E}(Q)[t]=1$.

By observing figure 3*b*,*c*, we know that properties (1)–(5) above hold similarly for networks of both the small-world and the ER models. We thus have:

*Convergence principle*. For homogeneous networks of the grid model, the small-world model and the ER model, for every *r*, there is a threshold *γ*_0_, which is approximately $\arccos\sqrt{\left(1+r)/(1+2r\right)}$ (determined by our Nash equilibrium principle of the quantum PD game), with a slight perturbation by the random variations of the evolutionary games on the networks, such that, for any *γ*, if *γ* is slightly larger than *γ*_0_, then there is a small number *T*, such that, for any evolution E, and any *t*≥*T*, the equilibrium frequency of super-cooperators of the quantum PD games on the network at step *t* is $\rho_{G}^{E}(Q)[t]=1-\epsilon$, where *ϵ* is either 0 or some small number determined by the perturbation of random variations of the evolutionary quantum games on the networks.

At the same time, the convergence principle above satisfies the following:

*Perturbation phenomenon of convergence.* Both the thresholds for convergence and the equilibrium frequencies of the converged strategy are perturbed by the random variations of the structures of the networks. For example, by observing figure 3*a*–*c*, we know that, for networks of the grid and the small-world models, the equilibrium frequency of super-cooperators is $\rho^{E}(G)=1$, and for random networks of the ER model, the equilibrium frequency of super-cooperators is $\rho^{E}(G)=1-\epsilon$ for some small number *ϵ*, and that the thresholds *γ*_0_ for the convergence of super-cooperation for different models are slightly perturbed by the random variations of the different structures of the networks.

This establishes the full convergence principle of super-cooperation in evolutionary quantum PD games on homogeneous networks.

## 7. Phase-transition phenomenon

In §3, we have shown that super-cooperation converges in evolutionary quantum PD games in homogeneous networks on the grid, the small-world and the ER models. In this section, we will show that there is a phase transition from the convergence of defection to the convergence of super-cooperation of the evolutions of quantum PD games on homogeneous networks. As before, we consider the networks of the grid, the small-world and the ER models. We study the curves of *ρ*(*C*), *ρ*(*D*) and *ρ*(*Q*) simultaneously. The experiments here for *ρ*(*C*), *ρ*(*D*) and *ρ*(*Q*) are the same as those in §3.

**Figure 4.**
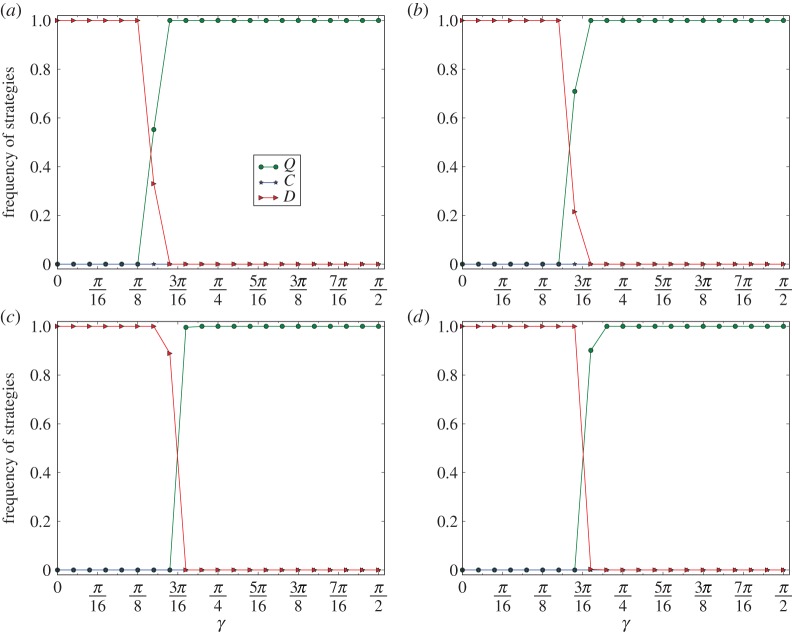
Phase transition from the convergence of defection to the convergence of super-cooperation on grid graphs. The equilibrium frequencies in the figure are the least ratios of the *C*-, *D*- and *Q*-strategies of the last 5000 steps of 10 000 steps of 10 evolutions of the grid network. (*a*–*d*) Correspond to the equilibrium frequencies of the games for *r*=0.4, 0.6, 0.8 and 1, respectively.

In figure 4, we depict the curves of *ρ*(*C*), *ρ*(*D*) and *ρ*(*Q*) on a grid of 100×100 for 10 evolutions of the quantum PD games. Figure 4*a*–*d* corresponds to the curves for *r*=0.4, 0.6, 0.8 and 1, respectively.

By observing figure 4, we have the following results. (1) For *r*=0.4. If *γ* is slightly less than 5*π*/32, then *ρ*(*D*)=1, and if *γ* is slightly larger than 5*π*/32, then *ρ*(*Q*)=1. (2) For *r*=0.6. If *γ* is slightly less than 3*π*/16, then *ρ*(*D*)=1, and if *γ* is slightly larger than 3*π*/16, then *ρ*(*Q*)=1. (3) For *r*=0.8. If *γ* is slightly less than 3*π*/16, then *ρ*(*D*)=1, and if *γ* is slightly larger than 3*π*/16, then *ρ*(*Q*)=1. (4) For *r*=1. If *γ* is slightly less than 13*π*/64, then *ρ*(*D*)=1, and if *γ* is slightly larger than 13*π*/64, then *ρ*(*Q*)=1.

By (1)–(4), we have that, for any *r*, for $\gamma_{0}\approx \arccos\sqrt{\left(1+r)/(1+2r\right)}$, and for any *γ*, if *γ* is slightly less than *γ*_0_, then for any evolution E the equilibrium frequency of the defectors *ρ*(*D*) is 1, and if *γ* is slightly larger than *γ*_0_, then for any evolution E the equilibrium frequency of the super-cooperators is *ρ*(*Q*)=1. Therefore, there is a phase transition from the convergence of defection to the convergence of super-cooperation in evolutionary quantum PD games on grid graphs.

**Figure 5.**
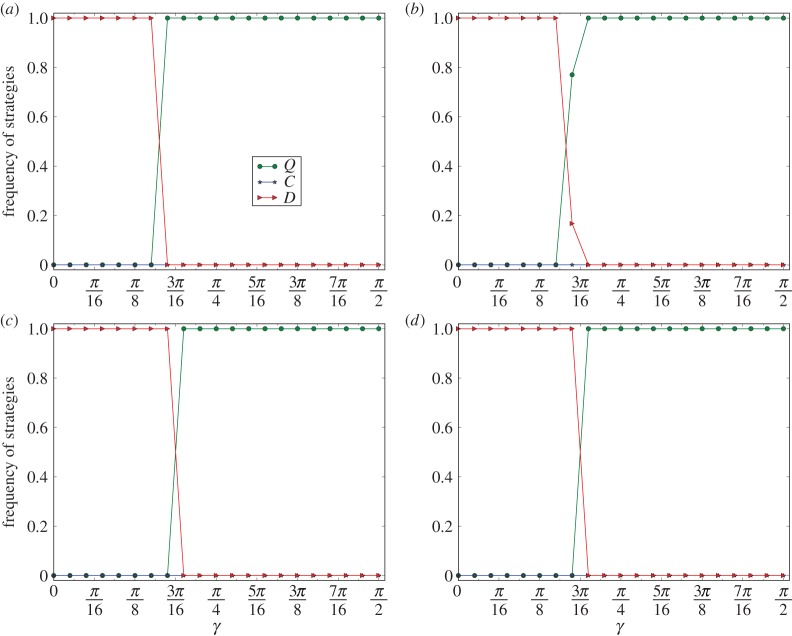
Phase transition from convergence of defection to convergence of super-cooperation on networks of the small-world model. The type of the network is: number of nodes *n*=10 000, average degree *d*=4 and *p*=0.01. The equilibrium frequencies of the figure are the least ratios of the *C*-, *D*- and *Q*-strategies of the last 5000 steps of 10 000 steps for 10 evolutions of each of 10 networks of the same type of the small-world model. (*a*–*d*) Correspond to the equilibrium frequencies of the games for *r*=0.4, 0.6, 0.8 and 1, respectively.

In figure 5, we depict the curves of *ρ*(*C*), *ρ*(*D*) and *ρ*(*Q*) on networks of the two-dimensional small-world model [26]. The type of the network includes: the number of nodes *n*=10 000, average degree *d*=4, with *p*=0.01. The curves are the minimal equilibrium frequencies among 10 evolutions of each of 10 networks. The curves in figure 5*a*–*d* correspond to the cases of *r*=0.4, 0.6, 0.8 and 1, respectively.

By observing figure 5, we have that the properties (1)–(4) for the grid graphs hold similarly for networks of the small-world model.

**Figure 6.**
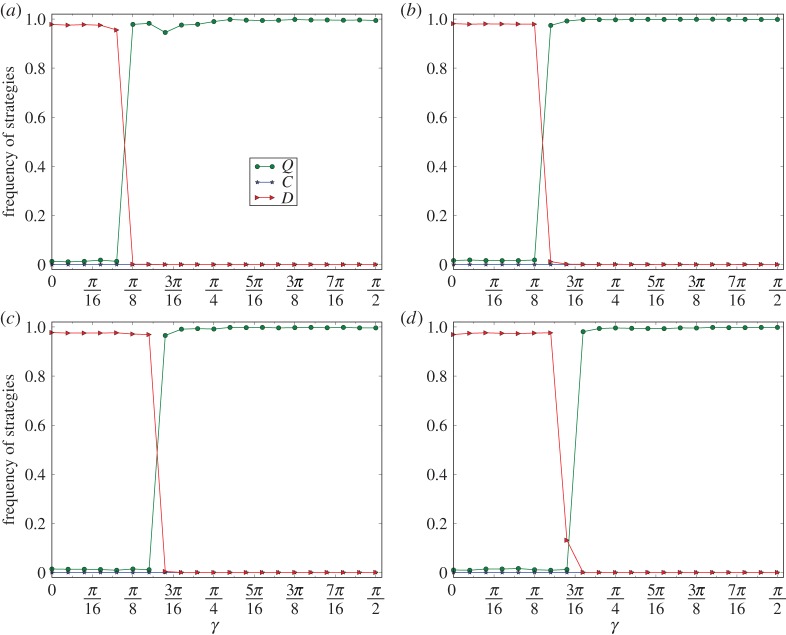
Phase transition from the convergence of defection to the convergence of super-cooperation on networks of the ER model with average degree *d*=4. The equilibrium frequencies of the figure are the least ratios of the *C*-, *D*- and *Q*-strategies of the last 5000 steps of 10 000 steps for 10 evolutions of each of 10 networks of the same type of the small-world model. (*a*–*d*) Correspond to the curves for *r*=0.4,0.6,0.8 and 1, respectively.

In figure 6, we depict the curves of *ρ*(*C*), *ρ*(*D*) and *ρ*(*Q*) on networks of the ER model [25]. The type of the network is: number of nodes *n*=10 000 and average degree *d*=4. The curves are the minimal equilibrium frequencies among 10 evolutions of each of 10 networks of the same type. The curves in figure 6*a*–*d* correspond to the cases of *r*=0.4, 0.6, 0.8 and 1, respectively.

By observing figure 6, we have that, for each *r*, for $\gamma_{0}\approx \arccos\sqrt{\left(1+r)/(1+2r\right)}$, and for any *γ*, if *γ* is slightly less than *γ*_0_, then for any *t*>5000, and for any evolution E, $\rho_{G}^{E}(D)[t]\approx 1$, and if *γ* is slightly larger than *γ*_0_, then for any *t*>5000, and for any evolution E, $\rho_{G}^{E}(Q)[t]\approx 1$.

By observing figures 4–6, we have the following *phase transition*: there is a phase transition from the convergence of defection to the convergence of super-cooperation in evolutionary quantum PD games on homogeneous networks, for which the thresholds of phase transitions are principally given by $\gamma =\arccos\sqrt{\left(1+r)/(1+2r\right)}$ with a slight perturbation by the structures of the networks.

At the same time, we have that the phase-transition phenomenon satisfies the following *perturbation*: the thresholds for the phase transition are principally determined by our Nash equilibrium principle, and simultaneously perturbed by the variations of the structures of the networks. For example, by comparing figures 4–6, we have that the thresholds for the phase transitions for the grid graphs and networks of the small-world model are almost identical, and that the thresholds for the phase transitions for the networks of the ER model are slightly smaller than the corresponding thresholds for the networks of the small-world model.

Finally, we remark that the increase in size *n* of the networks never changes the phase transitions of the evolutionary games. However, if the size *n* is trivially small, the phase transition may be affected. Therefore, our results hold for all non-trivially large networks.

## 8. Phase-transition principle

In §7, we have shown that there is a phase transition from the convergence of defection to the convergence of super-cooperation in the evolutions of quantum PD games on the homogeneous networks of the grid, the small-world and the ER models, and that the threshold for the phase transition is determined to be $\arccos\sqrt{\left(1+r)/(1+2r\right)}$ by our new Nash equilibrium principle with a slight perturbation determined by the structures of the networks.

In this section, we will show that both convergence and phase transition occur quickly, that the phase transitions are clear-cut jumping from 0 to 1, and that our Nash equilibrium principle explores an equilibrium belt for coexistence of defectors and super-cooperators.

**Figure 7.**
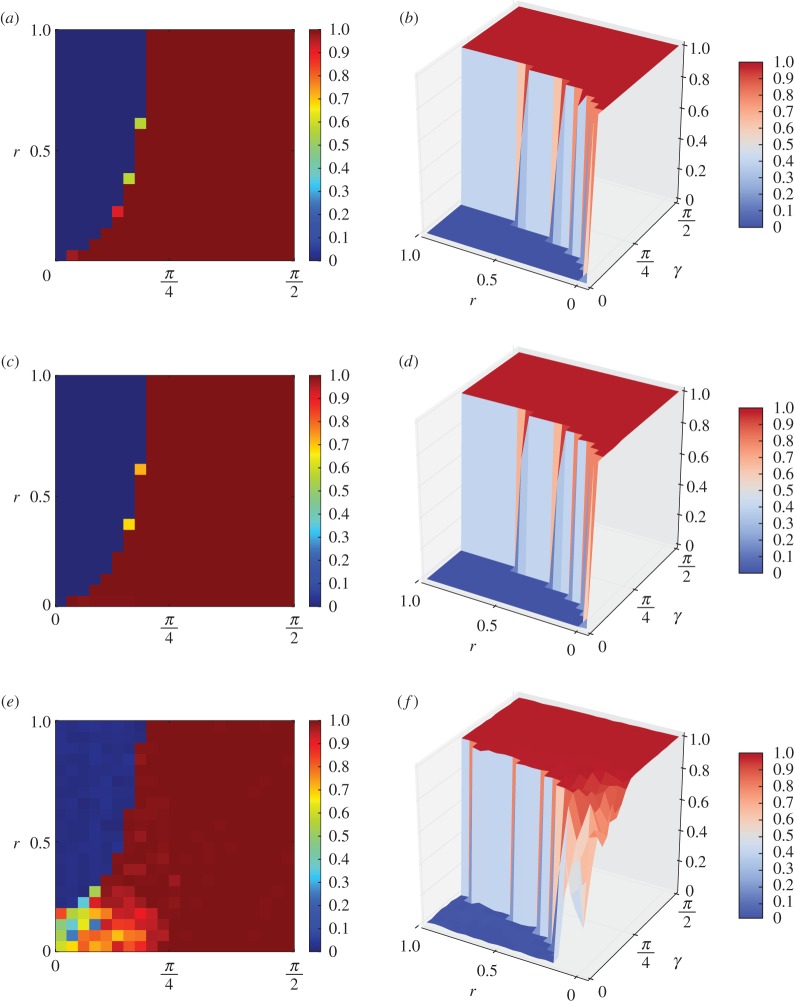
Colour belt for phase transition and for the coexistence of defectors and super-cooperators for evolutions of quantum PD games on networks of the grid, small-world and the ER models. Colour 0 means that *ρ*(*Q*)=0 and *ρ*(*D*)=1, and colour 1 means that *ρ*(*Q*)=1 and *ρ*(*D*)=0. The unit for *r* is $\frac{1}{20}$, and the unit for *γ* is *π*/64. Panels (*a*) and (*b*) are for a grid graph; (*c*) and (*d*) are for the small-world model; and (*e*) and (*f*) are for the ER model.

In figure 7*a*–*f*, we give two- and three-dimensional curves of the colour bars for the colours of evolutions of the quantum PD games on networks of the grid, small-world and ER models, respectively. Figure 7*a*,*b* shows the two- and three-dimensional representations of the colours from 0 (denoting *D*) to 1 (denoting *Q*) for the grid of 100×100, respectively. Figure 7*c*,*d* shows the two- and three-dimensional representations of the colours from *D* to *Q* for networks of the small-world model, respectively. Figure 7*e*,*f* shows the two- and three-dimensional representations of the colours from *D* to *Q* of networks of the ER model, respectively. Figure 7*a*,*c*,*e* shows the two-dimensional colours of *ρ*(*Q*) of the games for 20×32 choices of (*r*,*γ*), where the unit for *r* is $\frac{1}{20}$, and the unit for *γ* is *π*/64. Figure 7*b*,*d*,*f* shows the three-dimensional colours of *ρ*(*Q*) of the games for 20×32 choices of (*r*,*γ*), where the unit for *r* is $\frac{1}{20}$, and the unit for *γ* is *π*/64. In figure 7, colour 0 means that *ρ*(*Q*)=0, in which case *ρ*(*D*)=1, and colour 1 means that *ρ*(*Q*)=1, in which case *ρ*(*D*)=0, where *ρ*(*Q*) and *ρ*(*D*) are the least ratios of the *Q*- and *D*-strategy nodes of the last 100 steps of evolutionary games of 500 steps for 10 evolutions for each of 10 networks of the same type, with an exception for the grid model, for which a type has only one network.

According to figure 7*a*,*c*,*e*, the classical PD that is coded by *γ*=0 fails to converge to the super-cooperation, and the pure quantum game corresponding to *γ*=*π*/2 converges to super-cooperation. However, our game contains rich information about the role of the varying *γ*.

By observing figure 7, we have the following *phase-transition principle*. (1) In each case, there is a phase transition with either a phase-transition curve or a phase-transition belt along the curve given by $\gamma =\arccos\sqrt{\left(1+r)/(1+2r\right)}$. For example, by figure 7*a*,*c*, there is a phase-transition curve for networks of the grid and the small-world models, and by figure 7*e*, there is a narrow phase-transition belt for networks of the ER model. (2) By figure 7*b*,*d*, we have that for the grid graph and networks of the small-world model, there is a quick phase transition along the boundary of thresholds given by $\gamma =\arccos\sqrt{\left(1+r)/(1+2r\right)}$, such that if *γ* is slightly less than $\arccos\sqrt{\left(1+r)/(1+2r\right)}$ then the equilibrium frequency of defectors quickly converges to 1, and if *γ* is slightly larger than $\arccos\sqrt{\left(1+r)/(1+2r\right)}$ then the equilibrium frequency of super-cooperators quickly converges to 1. (3) By figure 7*e*,*f*, for the networks of the ER model, property (2) above principally holds, except for a small area close to the zero point (0,0). In this case, if (*r*,*γ*) is close to (0,0), then the phase transition is perturbed by the random variations of the structures of the random networks of the ER model. (4) By figure 7, we have that the curve $\gamma \approx \arccos\sqrt{\left(1+r)/(1+2r\right)}$ by our Nash equilibrium principle determines an equilibrium boundary for the coexistence of two competing species, beyond which one species may conquer the other. This is also an interesting discovery which explores the principle of coexistence of divergent species. (5) By figure 7, in any case, both the convergence of either defection or super-cooperation and the phase transition from the convergence of defection to the convergence of super-cooperation occur in a small number of steps of the evolutionary games; in fact, within the first 400 steps of the evolutionary games in our experiments.

The results in (1)–(5) above also demonstrate that the phase-transition principle is always accompanied by a perturbation principle.

The phase-transition phenomenon explored in (1)–(5) above has significant implications. (i) In a physical system, even if entanglement exists, it is hard to ensure that the entanglement degree is *γ*=*π*/2. Our results show that slightly non-trivial entanglement is sufficient to change the physical system to a desirable global stability, and that trivial entanglement, i.e. below the phase-transition point, does not change the global state of the physical system. (ii) Owing to the fact that the implementation of our game is classical, and that there is an entangled relationship between the players in the real-world games, our game can be regarded as a well-defined classic game. This implies that the game may provide a theory for us to better understand the real games in Nature and society.

## 9. Perturbation principle of convergence and phase transition of evolutionary quantum PD games

We have seen that there is a perturbation phenomenon accompanying both the convergence principle and the phase-transition principle.

By observing figures 3–7, we have the following *perturbation principle*. (1) The fundamental reason for perturbation is the variations in the structures of the networks. (2) Perturbation changes the three measures of the thresholds of phase transition, the width of the phase-transition belt, and the equilibrium frequencies of the converged strategy.

The reasons for this are as follows. By comparing figure 3*a*–*c*, we have that the convergent equilibrium frequencies of super-cooperators are either 1 or ≈1. This is the result of perturbation by the different structures of the networks. By comparing figures 4–6, both the convergent equilibrium frequencies of the strategies and the thresholds for phase transitions are slightly perturbed by the different structures of the networks. For example, the convergent equilibrium frequencies of the strategies are usually 1 for networks of the grid and the small-world models, and are usually ≈1 for networks of the ER model, and the thresholds for networks of the ER model are slightly smaller than those of the corresponding networks of the small-world model, and of the grid model. By comparing figure 7*a*–*f*, we have that the phase-transition phenomena of evolutionary quantum games on networks of the ER model are perturbed by the different structures of the networks due to the random variations of structures of the networks generated by the model. For example, when *γ* and *r* are close to 0, the random variations of the structures of the ER model radically perturb the convergent strategies and the phase-transition phenomena, as we observed from figure 7*e*,*f*.

These results demonstrate that the convergence and phase transition of strategies are both principally determined by our Nash equilibrium principle, with a perturbation by variations of structures of networks.

## 10. Divergence and emergence of super-cooperation of evolutionary quantum games on heterogeneous networks

In this section, we study the convergence of evolutionary quantum PD games on the heterogeneous networks of the PA model [7].

**Figure 8.**
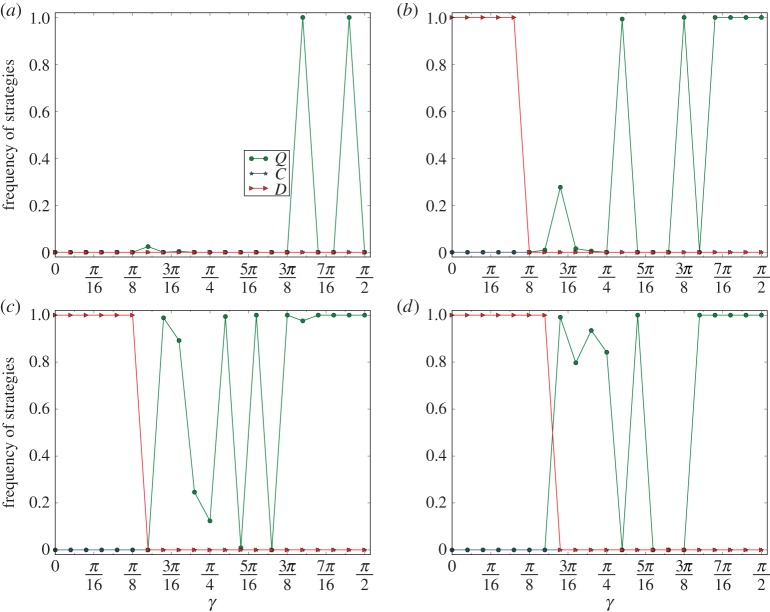
Divergence of super-cooperation on networks of the PA model with average degree *d*=4. The equilibrium frequencies in this figure are the least ratios of the *C*-, *D*- and *Q*-strategies of the last 5000 steps of 10 000 steps of 10 evolutions of each of 10 networks of the same type. (*a*–*d*) Correspond to the curves for *r*=0.4,0.6,0.8 and 1, respectively.

In figure 8, we depict the curves of *ρ*(*X*) for *X*∈{*C*,*D*,*Q*} on networks of the PA model with number of nodes *n*=10 000, and average number of edges *d*=4. The experimental method here is the same as that in §7.

According to figure 8, we have the following *divergence phenomenon*. For any *r* and any *γ*, super-cooperation fails to converge in evolutionary quantum PD games on heterogeneous networks of the PA model with edge parameter *d* small. (It is an interesting open question to investigate the convergence/divergence problem for the networks of the PA model for varying *d*. We conjecture that convergence occurs for the networks with large *d*. This means that the networks of the PA model have dramatically different structures for small *d* and large *d*.)

The result is in sharp contrast to that of the emergence of super-cooperation of evolutionary quantum PD games on the heterogeneous networks of the PA model in [27]. For any *r*, and for a threshold *γ*_0_ approximately equal to $\arccos\sqrt{(1+r)/(1+2r})$, if *γ* is slightly less than *γ*_0_, then the average equilibrium frequency of the defectors is ≈1, and if *γ* is slightly larger than *γ*_0_, then the average equilibrium frequency of the super-cooperators is ≈1. Clearly, the emergence principle is accompanied by a perturbation phenomenon with the random variations of the structures of the networks. As before, perturbation may change the thresholds for phase transition, the widths of phase transition and the equilibrium frequencies of the emerged strategies of evolutionary quantum games on heterogeneous networks of the PA model.

## 11. Conclusion and discussion

We propose the notion of convergence of evolutionary games on networks, and investigate the convergence of evolutionary quantum PD games on networks of the classical models. Our theory explores a number of new principles of evolutionary quantum PD games on networks. We found a Nash equilibrium principle, a dynamics principle of evolutionary quantum games, a convergence principle of evolutionary quantum PD games on homogeneous networks, a phase-transition principle of evolutionary quantum PD games on homogeneous networks, a divergence phenomenon of evolutionary quantum games on the heterogeneous networks of the PA model with small *d* and an emergence principle of evolutionary quantum games on heterogeneous networks. We also found that the convergence principle, the phase-transition principle for homogeneous networks and the emergence principle for heterogeneous networks are all determined by our Nash equilibrium principle given by a phase-transition point $\gamma =\arccos\sqrt{\left(1+r)/(1+2r\right)}$ with an accompanying perturbation phenomenon determined by variations of the structures of the networks. Our results imply that the stochastic process of the evolutionary games of an interacting and competing system may converge to a stable state determined by the phase-transition point of the local property of the degree of the entangled relationship among the individuals. In particular, our theory implies that a slightly non-trivial entangled relationship of the many bodies guarantees a stable and global property of highly complex stochastic processes that may occur in a wide range of disciplines such as physics, chemistry, economics, society and even biology. This result may have implications for the establishment of a new type of international relationship in the current highly connected small world, which differs from the old world based on classic games such as PD and zero-sum games, etc.

We note that, for the classical PD game, in the homogeneous networks cooperation fails to emerge, in the heterogeneous networks of the PA model with small *d* cooperation emerges, and in the networks of the PA model with large *d* cooperation fails to emerge. These results complement our convergence results for super-cooperation. This observation implies an interesting hypothesis that, for a graph *G*, either cooperation emerges for the classical PD games or super-cooperation converges for our game, leading to future studies in evolutionary game theory. Therefore, convergence could be an accompanying notion of emergence, both of which are essential for evolutionary game theory. The fundamental challenge in the theory is hence clearly the following: to find the measures of graphs that guarantee the emergence of cooperation in classic games, and the measures that guarantee the convergence of super-cooperation for our game. Remarkably, our results imply that the convergence results of super-cooperation for our game in the homogeneous networks could be mathematically established, which calls for a new method for analysing the stochastic process of evolutionary games in a network.

Finally, we note that Du *et al.* [31] has realized experimentally the quantum game of two players on a nuclear magnetic resonance quantum computer. Our results realize the many-body quantum game by a classical device. The progress here suggests that it would be very interesting to realize our many-body quantum game by quantum computers.
